# Regulation of multidrug resistance 1 expression by CDX2 in ovarian mucinous adenocarcinoma

**DOI:** 10.1002/cam4.697

**Published:** 2016-04-06

**Authors:** Iemasa Koh, Takao Hinoi, Kazuhiro Sentani, Eiji Hirata, Suguru Nosaka, Hiroaki Niitsu, Masashi Miguchi, Tomohiro Adachi, Wataru Yasui, Hideki Ohdan, Yoshiki Kudo

**Affiliations:** ^1^ Program for Applied BiomedicineDivision of Clinical Medical ScienceDepartment of Obstetrics and GynecologyGraduate School of Biomedical ScienceHiroshima UniversityHiroshima734‐8551Japan; ^2^ Program for Biomedical ResearchDivision of Frontier Medical ScienceDepartment of Gastroenterological and Transplant SurgeryGraduate School of Biomedical ScienceHiroshima UniversityHiroshima734‐8551Japan; ^3^ Department of Molecular PathologyInstitute of Biomedical and Health SciencesHiroshima UniversityHiroshima734‐8551Japan

**Keywords:** CDX2, chemotherapy, MDR1, mucinous adenocarcinoma, ovarian cancer

## Abstract

Epithelial ovarian cancer is an aggressive gynecological malignancy with a high mortality rate. Resistance against chemotherapeutic agents often develops in ovarian cancer patients, contributing to high recurrence rates. The multidrug resistance 1 (*MDR1/ABCB1*) gene encodes P‐glycoprotein, which affects the pharmacokinetic properties of anticancer agents. We previously reported that the Caudal‐related homeobox transcription factor CDX2 transcriptionally regulates *MDR1* expression in colorectal cancer. CDX2 is a factor that influences cancer cell differentiation, malignancy, and cancer progression. We hypothesized that profiling of CDX2 and MDR1 expression could be an effective strategy for predicting anticancer drug resistance. We studied the expression of these factors in clinical samples from ovarian cancer patients. We found that endogenous *MDR1* expression was positively associated with CDX2 expression in ovarian mucinous adenocarcinoma. Using ovarian mucinous adenocarcinoma cell lines, we also observed decreased *MDR1* expression following inhibition of CDX2 by RNA interference. In addition, CDX2 overexpression in MN‐1 cells, which display low endogenous CDX2, resulted in upregulation of *MDR1* expression. CDX2 induced MDR1‐dependent resistance to vincristine and paclitaxel, which was reversed by treatment with the MDR1‐specific inhibitor verapamil. Our findings show that CDX2 promotes upregulation of *MDR1* expression, leading to drug resistance in ovarian mucinous adenocarcinoma. Therefore, our study demonstrates the potential of novel chemotherapy regimens based on CDX2 status and MDR1 expression in ovarian mucinous adenocarcinoma.

## Introduction

Ovarian cancer is the most lethal form of gynecological malignancy. Treatment of epithelial ovarian cancers (EOCs) is based on a combination of surgery and chemotherapy. However, the development of resistance against chemotherapeutic agents following initial treatment and cases of disease recurrence are frequent in ovarian cancer. EOCs were historically thought to arise from ovarian surface epithelial cells, with metaplasia leading to four major histological types: serous, endometrioid, clear cell, and mucinous. Ovarian mucinous adenocarcinoma is the third most common type, comprising approximately 10% of all primary EOCs 1. Currently, combination chemotherapy consisting of carboplatin and paclitaxel is the standard protocol for initial treatment to EOCs, although ovarian mucinous adenocarcinoma is known to be resistant to this regimen 2, 3, 4, 5, 6. Ovarian mucinous adenocarcinoma is generally resistant to chemotherapeutic agents. Poorly differentiated mucinous adenocarcinoma shows a resistance to chemotherapeutic agents, but it is important to recognize that well‐differentiated types also display resistance. Despite the fact that mucinous adenocarcinoma is often diagnosed at an early stage, this histological type has a poorer prognosis compared with that of other histological subgroups 2, 3, 4. Since chemosensitivity is one of the main prognostic factors for patients with advanced EOCs, a low response to conventional platinum‐ and taxane‐based chemotherapy is associated with a poor prognosis in mucinous adenocarcinoma 2, 3, 4.

The *MDR1* gene, initially cloned by Roninson and others in 1986, encodes P‐glycoprotein, also known as multidrug resistance 1 (MDR1/ABCB1), a member of the ATP‐binding cassette (ABC) family 7, 8. The role of MDR1 is clinically significant, as this factor not only confers multidrug resistance, but also affects the pharmacokinetic properties of various drugs 7. The MDR1 protein has 12 transmembrane domains and two ATP‐binding domains. *MDR1* is overexpressed in drug‐resistant cancer cells, suggesting this gene and its product, P‐glycoprotein, play a critical role in drug resistance 7, 9, 10. MDR1 confers multidrug resistance to cancer cells by promoting cellular excretion of structurally diverse chemotherapeutic compounds, such as vinca alkaloid, anthracycline, and taxane 7.

We previously demonstrated that endogenous *MDR1* expression correlates with expression of the intestine‐specific *caudal*‐related homeobox transcription factor CDX2, and that *MDR1* is a direct transcriptional target of CDX2 in various colorectal cancer cell lines and human colon cancer tissue 11. CDX2 expression is observed in the epithelium of the small intestine and colon, as well as in well‐differentiated colon carcinoma but not in poorly differentiated colon carcinoma 12, 13. Moreover, aberrant CDX2 expression was detected in metaplasia of the stomach 14. Taken together, these findings suggest that CDX2 has critical functions in intestinal development and maintenance of the intestinal phenotype 12, 15, 16. In addition, these findings indicate that CDX2 influences the degree of differentiation, malignancy, and cancer progression in the colon and stomach 12, 13, 14. Since CDX2 regulates *MDR1* expression, we hypothesized that these two factors could be useful markers for predicting drug resistance to anticancer agents in the stomach and possibly other organs 11.

In gynecological cancer, immunohistochemical detection of CDX2 can be a useful marker to distinguish metastatic colorectal carcinoma involving the ovary from primary ovarian mucinous adenocarcinoma 17, 18. However, high CDX2 expression was observed in ovarian mucinous adenocarcinoma, of which 64% of cases were positive 19. In ovarian mucinous adenocarcinoma, no reports have described an association between the expression of CDX2 and MDR1 and the degree of cancer cell differentiation.

Here, we studied the expression of CDX2 and MDR1 in clinical EOC tissue samples. In addition, we investigated *MDR1* regulation by CDX2 and MDR1‐associated drug resistance in ovarian mucinous adenocarcinoma cells.

## Materials and Methods

### Plasmids

A full‐length, wild‐type CDX2 cDNA was amplified by PCR using hexamer‐primed cDNA from normal human colon tissue as a template. The *CDX2* gene was inserted into the multiple cloning site of the retroviral expression vector pPGS‐CMV‐CITE‐neo (pPGS‐neo, provided by G. Nabal, NIH, Bethesda, MD) to generate pPGS‐CDX2.

### Clinical tissues and immunohistochemistry

A total of 53 epithelial ovarian cancer cases were diagnosed and resected in our facility between 2005 and 2013. Formalin‐fixed, paraffin‐embedded (FFPE) tissues were stained using the avidin‐biotin complex method, as previously described 14. Rabbit monoclonal anti‐CDX2 (clone EPR2764Y; NICHIREI, Tokyo, Japan) and mouse monoclonal anti‐MDR1 (clone C494; COVANCE, Dedham, MA) antibodies were used at 1:1 and 1:200 dilution, respectively. In accordance with the Ethical Guidelines for Human Genome/Gene Research enacted by the Government of Japan, tissue specimens were collected and used following approval by the Ethical Review Committee of the Hiroshima University School of Medicine and by the ethical review committees of collaborating organizations.

### Cell lines and cell culture

The five ovarian mucinous adenocarcinoma cell lines used in this study (MCAS, RMUG‐S, MN‐1, OMC‐1, OMC‐3) were obtained from the following sources: MN‐1 from Scienstuff Co. Ltd (Nara, Japan) 20, OMC‐1 from Dr. Tsuyoshi Saito (School of Medicine, Sapporo Medical University, Sapporo, Japan) 21, MCAS and RMUG‐S from the Japanese Collection of Research Bioresources (JCRB) Cell Bank, and OMC‐3 from RIKEN Bio Resource Center Cell Bank (Institute of Physical and Chemical Research, Tsukuba, Japan). The DLD‐1 colorectal cancer cell line used as a positive control was obtained from the American Type Culture Collection. MCAS cells were grown in Eagle's minimal essential medium (Wako, Osaka, Japan) with 20% fetal bovine serum (FBS), 100 units/mL penicillin, and 100 *μ*g/mL streptomycin (Invitrogen, Carlsbad, CA). MN‐1 cells were grown in Dulbecco's minimal essential medium (Wako) with 10% FBS and penicillin/streptomycin. RMUG‐S and OMC‐3 cells were grown in Ham's F12 medium (Wako) with 10% FBS and penicillin/streptomycin. OMC‐1 cells were grown in Minimum essential medium *α* medium (Wako) with 10% FBS and penicillin/streptomycin. All cell lines were maintained at 37°C in 5% CO_2_.

### Retrovirus infection

The amphotropic Phoenix packaging cell line provided by G. Nolan (Stanford University, Stanford, CA) was propagated in Dulbecco's minimal essential medium and transfected with retroviral expression constructs using FuGENE6 (Promega, Madison, WI), as previously described 14. Briefly, cells were seeded in 60 mm dishes 40 h before transfection and were transfected with 2 *μ*g of retroviral plasmid DNA and 12 *μ*L of FuGENE6. After 24 h, the growth medium was replaced. Then, 48 h after transfection, the supernatant containing nonreplicating amphotropic virus was harvested and filtered 14. MN‐1 cells in 60 mm dishes at 70–80% confluency were infected with virus supernatant and fresh medium at a ratio of 1:1 in the presence of 4 *μ*g/mL polybrene (Sigma, Milwaukee, WI). After 24 h of incubation with the virus, MN‐1 cells were subcultured at split ratios of 1:5 to 1:10 and selected for 2–3 weeks in media containing 1 mg/mL G418 (Wako). Real‐time PCR and Western blot were used to examine CDX2 expression in MN‐1 cells stably expressing *CDX2* (MN‐1/PGS‐CDX2) and control cells (MN‐1/PGS‐neo) maintained in Dulbecco's minimal essential medium supplemented with G418 at a concentration of 400 *μ*g/mL.

### Quantitative real‐time reverse transcription‐PCR

Total RNA was isolated using an RNeasy Mini Kit (Qiagen, Valencia, CA) with DNase I (Sigma) treatment; reverse transcription (RT) was performed using an Omniscript RT Kit (Qiagen), as described in the manufacturer's protocol. Total RNA (1 *μ*g) was used for cDNA synthesis. Real‐time RT‐PCR was performed using an ABI 7300HT with Power SYBR Green PCR Master Mix (Applied Biosystems, Foster, CA) and sequence‐specific primers, as indicated in Table 1.

**Table 1:** Real‐time PCR primers

Gene name	Forward primer 5′‐3′	Reverse primer 5′‐3′	Fragment size
*CDX2*	GAACCTGTGCGAGTGGATG	GGTGATGTAGCGACTGTAGTGAA	148 bp
*MDR1*	GTCCCAGGAGCCCATCCT	CCCGGCTGTTGTCTCCATA	70 bp

### Western blot

Western blot analysis was performed as previously described 14. Anti‐CDX2 mouse monoclonal (clone CDX2‐88, BioGenex Laboratories, Inc. Fremont, CA), and anti‐human MDR1 monoclonal antibodies (clone C219, Calbiochem, San Diego, CA) were used at 1:100, and 1:50 dilutions, respectively. The membrane was stripped and reprobed with anti‐GAPDH antibodies (clone 6C5; Santa Cruz Biotechnology, Inc. Dallas, TX) to verify loading and transfer.

### RNA interference

Two small interfering RNA (siRNA) duplexes targeting *CDX2* mRNA (5′‐AACCAGGACGAAAGACAAAUA‐3′, CDX2 siRNA; and MISSION endoribonuclease‐prepared siRNA, *CDX2* esiRNA) and a nonsilencing siRNA duplex (MISSION siRNA Universal Negative Control SIC‐001) were synthesized by Qiagen‐Xeragon (Huntsville, AL). MISSON esiRNA is an endoribonuclease‐prepared siRNA pool comprised of a heterogeneous mixture of siRNAs that all target the same mRNA sequence. Cells were cultured in antibiotic‐free medium for 24 h before transfection and were then transfected with siRNA (200 pmol) using RNAiMAX (Invitrogen). Silencing was confirmed 72 h after transfection. For validation, quantitative RT‐PCR was performed.

### Cytotoxicity assay

Paclitaxel, doxorubicin, and carboplatin were obtained from Wako. Vincristine and verapamil were purchased from Sigma. An MTS cytotoxicity assay was performed to examine cell survival after exposure to chemotherapeutic agents. Cells were seeded at 3000 cells/100 *μ*L per well in 96‐well microtiter plates. After a 48‐h incubation period, cells were treated with a range of concentrations of each chemotherapeutic agent. To examine the effect of verapamil, a known MDR1 inhibitor 22, 1 *μ*mol/L was coadministered with each chemotherapeutic agent; a pilot experiment showed that this concentration was not cytotoxic to MN‐1/PGS‐CDX2 or MN‐1/PGS‐neo cells (data not shown). After 96 h, 20 *μ*L of MTS dye (1.9 mg/mL) was added to each well, and plates were incubated for 3 h. Absorbance in individual wells was determined at 490 nm using an ImmunoMini NJ‐2300 microplate reader (InterMed, Tokyo, Japan). Results were expressed as the concentration required to inhibit cell growth by 50% relative to that of nontreated control cells [IC50 (96 h)].

## Results

### Coexpression of CDX2 and MDR1 in human ovarian cancer tissue

**Figure 1:**
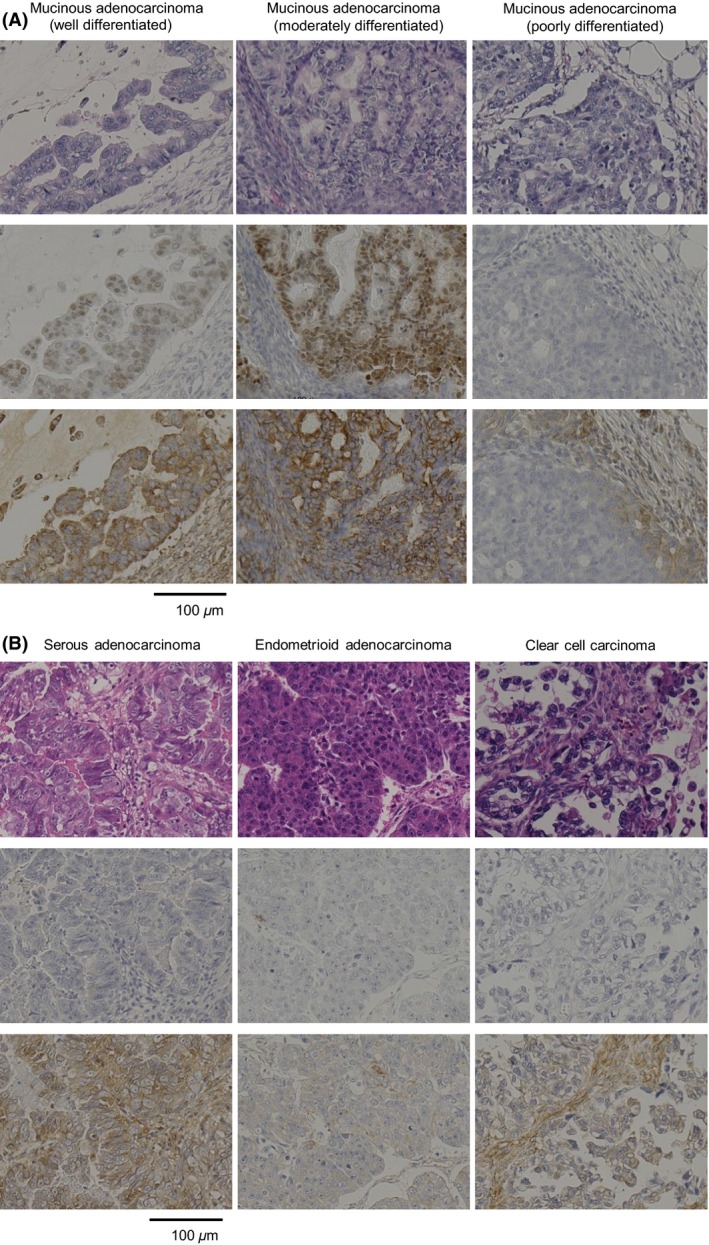
In ovarian cancer, particularly mucinous adenocarcinoma, expression of CDX2 and MDR1 correlated with the degree of cell differentiation. Immunohistochemistry was performed on formalin‐fixed, paraffin‐embedded tissues with hematoxylin and eosin (A and B, upper), with an anti‐CDX2 monoclonal antibody (A and B, middle), and with an anti‐MDR1 monoclonal antibody, C494 (A and B, lower) in (A) ovarian mucinous adenocarcinoma, (B) ovarian serous adenocarcinoma, endometrioid adenocarcinoma, and clear cell carcinoma tissue.

Immunohistochemical staining analysis revealed that expression of the CDX2 transcription factor was localized to nuclei, whereas expression of MDR1, encoding an ABC transporter, was localized to membranes (Fig. 1). Of 53 ovarian cancer cases studied, eight cases displayed positive CDX2 expression (15.1%) and 21 displayed positive MDR1 expression (39.6%) (Table 2). CDX2 expression was observed in ovarian mucinous adenocarcinoma with well‐ and moderately differentiated types, whereas expression was negative or faint in other histological types (Table 2, Fig. 1A and B). MDR1 expression was observed in well‐differentiated and moderately differentiated mucinous adenocarcinomas in all seven cases (100%); MDR1 expression was also detected in serous adenocarcinoma and clear cell carcinoma in five of 13 cases (38.5%) and six of 15 cases (40.0%), respectively. These immunohistochemical findings indicate that endogenous MDR1 expression is associated with CDX2 expression in human ovarian cancer tissue as well as mucinous ovarian cancer tissues (*P* < 0.001; in human ovarian cancer, and *P* < 0.05; in mucinous ovarian cancer, using Fisher's exact test) (Tables S1, S2).

**Table 2:** CDX2, MDR1 immunohistochemical staining in epithelial ovarian cancers

Histological typeDifferentiation degree (*n* = 53)		CDX2 (%)+	MDR1 (%)+
Ovarian mucinous adenocarcinoma (*n* = 14)	Well‐differentiated (*n* = 6)	6/6 (100)	6/6 (100)
	Moderately differentiated (*n* = 1)	1/1 (100)	1/1 (100)
	Poorly differentiated (*n* = 7)	0/7 (0)	2/7 (28.5)
Ovarian serous adenocarcinoma (*n* = 13)		0/13 (0)	5/13 (38.5)
Ovarian endometrioid adenocarcinoma (*n* = 11)		1/11 (9)	1/11 (9)
Ovarian clear cell carcinoma (*n* = 15)		0/15 (0)	6/15 (40)

Positive (+): >50% of tumor cells stained.

Well‐differentiated: Ratio of solid growth part 0–5%.

Moderately differentiated: Ratio of solid growth part 5–50%.

Poorly differentiated: Ratio of solid growth part 50–100%.

### Correlation between CDX2 and MDR1 expression in ovarian mucinous adenocarcinoma cells

**Figure 2:**
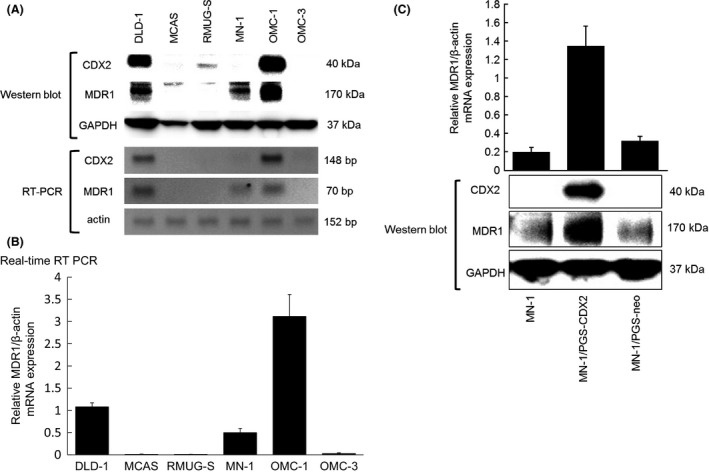
Expression of CDX2 and MDR1 in five ovarian mucinous adenocarcinoma cell lines. (A) In five ovarian mucinous adenocarcinoma cell lines (MCAS, RMUG‐S, MN‐1, OMC‐1, OMC‐3), Western blot analyses of MDR1 and CDX2 expression were performed using a mouse monoclonal antibody against human MDR1 (170 kDa) and a mouse monoclonal antibody against human CDX2 (40 kDa). RT‐PCR analysis of CDX2 and MDR1 expression was performed using specific CDX2 and MDR1 primers, respectively. A colorectal cancer cell line (DLD‐1) was used as a positive control. (B) Relative MDR1/beta‐actin mRNA expression in five ovarian mucinous adenocarcinoma cell lines and the DLD‐1 cell line using quantitative real‐time RT PCR. (C) Monoclonal anti‐CDX2 antibody detects the CDX2 protein in MN‐1/PGS‐CDX2 cells but not in MN‐1/PGS‐neo cells. Quantitative RT PCR and Western blot detects MDR1 transcripts and protein products, respectively, in MN‐1/PGS‐CDX2 cells; low MDR1 expression was observed in MN‐1/PGS‐neo cells.

We further investigated the correlation of CDX2 and MDR1 expression in ovarian mucinous adenocarcinoma cell lines. Western blot and RT‐PCR analyses revealed high expression of CDX2 mRNA and protein in parallel with high levels of MDR1 mRNA and protein in OMC‐1 cells; expression of CDX2 and MDR1 was not observed in MCAS, RMUG‐S, or OMC‐3 cells. In MN‐1 cells, MDR1 expression was weak, whereas CDX2 expression was not detected (Fig. 2A and B). None of the cell lines displaying weak or undetectable MDR1 expression displayed CDX2 protein expression.

MN‐1 cells showed very low endogenous CDX2 expression. To confirm whether CDX2 regulates *MDR1* expression in ovarian mucinous adenocarcinoma cells, we generated a polyclonal population of MN‐1 ovarian cancer cells expressing high levels of CDX2 by infecting these with replication‐defective retroviruses carrying a full‐length human *CDX2* cDNA 14. After expression of CDX2 protein was induced in MN‐1/PGS‐CDX2 cells, but not in MN‐1/PGS‐neo control cells, a robust induction of *MDR1* transcripts and protein in MN‐1/PGS‐CDX2 cells was confirmed by quantitative RT‐PCR and Western blot analyses, respectively (Fig. 2C).

### Downregulation of *MDR1* expression by inhibition of CDX2 using RNA interference in ovarian mucinous adenocarcinoma cells

**Figure 3:**
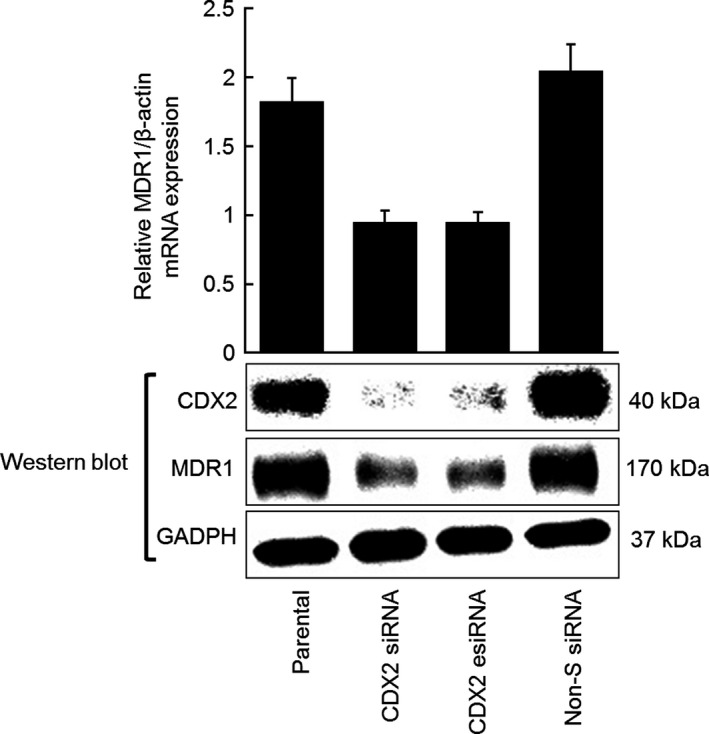
Inhibition of CDX2 expression by siRNA targeting leads to decreased MDR1 expression in the ovarian mucinous adenocarcinoma cell line OMC‐1. Assays were performed in triplicate; columns, mean; bars, SD.

To further confirm the regulation of *MDR1* expression by CDX2 in ovarian mucinous adenocarcinoma cells, we inhibited *CDX2* expression by RNA interference (RNAi) and determined the effect on *MDR1* transcript and protein expression. For this experiment, we used OMC‐1, an ovarian mucinous adenocarcinoma cell line with high endogenous expression of CDX2 and MDR1. CDX2‐specific siRNAs significantly suppressed CDX2 protein expression; *MDR1* transcript and protein levels were concomitantly downregulated by approximately 50% in OMC‐1 cells treated with CDX2 siRNAs compared with levels observed in parental and control siRNA‐treated cells (Fig. 3). These data indicate that CDX2 is involved in maintaining *MDR1* gene and protein expression in OMC‐1 cells.

### MN‐1 cells ectopically expressing CDX2 display MDR1‐dependent drug resistance

**Figure 4:**
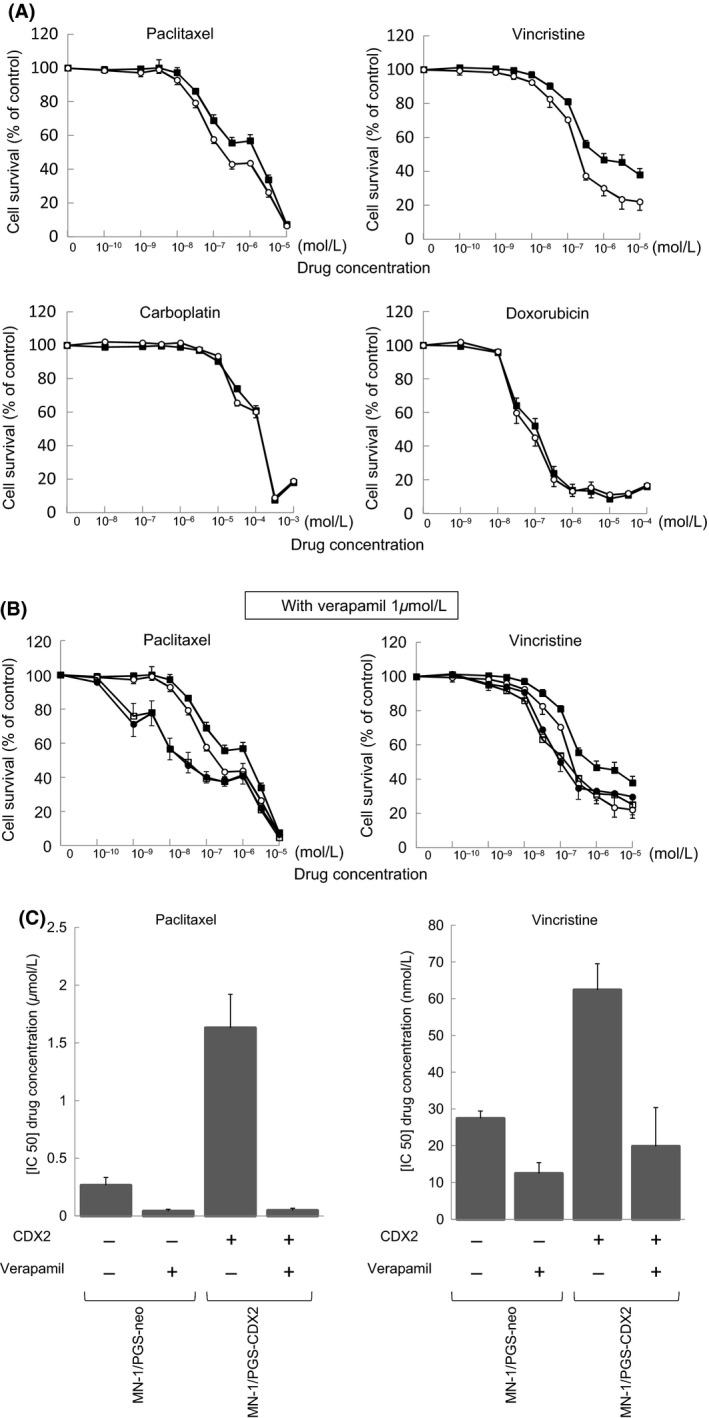
MN‐1 cells ectopically expressing CDX2 display MDR1‐dependent drug resistance. (A) Effect of chemotherapeutic drugs on MN‐1/PGS‐CDX2 (■) and MN‐1/PGS‐neo (○) cell lines. (B) Effect of additional verapamil on vincristine and paclitaxel in MN‐1/PGS‐CDX2 (□) and MN‐1/PGS‐neo (•) cell lines. (C) [IC50 (96 h)] determined by MTS assay on MN‐1/PGS‐CDX2 and MN‐1/PGS‐neo cells. Cotreatment with verapamil significantly recovered the sensitivity of vincristine and paclitaxel on MN‐1/PGS‐CDX2 cells. The cytotoxic assays were performed in triplicate; points, mean; bars, SD.

To determine whether MDR1 induced by CDX2 functions as a drug efflux pump, we analyzed the effects of various chemotherapeutic drugs on MN‐1/PGS‐CDX2 and MN‐1/PGS‐neo cells (Fig. 4A). Vincristine and paclitaxel, MDR1 substrates 23, displayed decreased cytotoxicity, 2.3‐ and 6.2‐fold increase in IC50 (96 h), respectively, in MN‐1/PGS‐CDX2 cells compared with MN‐1/PGS‐neo cells. However, the MDR1 nonsubstrates carboplatin and doxorubicin showed similar cytotoxicity in MN‐1/PGS‐CDX2 and MN‐1/PGS‐neo cells (Fig. 4A). To further examine MDR1‐dependent drug resistance, we repeated the assay in the presence of the MDR1 inhibitor verapamil. Cotreatment with 1 *μ*mol/L verapamil increased the cytotoxic activities of vincristine and paclitaxel in MN‐1/PGSCDX2 cells (Fig. 4B and C). Verapamil reduced the differences in drug‐induced cytotoxicity between MN‐1/PGS‐CDX2 and MN‐1/PGS‐neo cells (Fig. 4B and C). These results suggest that increased resistance to vincristine and paclitaxel in MN‐1/PGS‐CDX2 cells is associated with MDR1 upregulation induced by ectopic CDX2 expression.

## Discussion

In this study, we observed that CDX2 and MDR1 are expressed in ovarian mucinous adenocarcinoma and correlate with the degree of cancer cell differentiation. MDR1 upregulation by aberrant CDX2 expression could be related to drug resistance observed in ovarian mucinous adenocarcinoma, a conclusion arising from the following observations.

On the basis of an evaluation of clinical tissues of ovarian cancer cases, we observed overexpression of CDX2 in ovarian mucinous adenocarcinoma but only weak expression in other histological types, including serous adenocarcinoma, endometrioid adenocarcinoma, and clear cell carcinoma. Expression of CDX2 and MDR1 was most pronounced in well‐differentiated mucinous adenocarcinoma and weakest in poorly differentiated ones. These data suggest that CDX2 and MDR1 expression could be associated with the degree of cancer cell differentiation. Second, *MDR1* transcript expression and MDR1 protein expression were upregulated by CDX2 in MN‐1/PGS‐CDX2 cells, and MDR1 expression was concomitantly downregulated in OMC‐1 cells by CDX2 inhibition using RNAi. Additionally, increased resistance to chemotherapeutic drugs in MN‐1/PGS‐CDX2 cells was observed following *MDR1* overexpression. MDR1‐dependent drug resistance in MN‐1/PGS‐CDX2 cells was reversed by addition of the MDR1‐specific inhibitor verapamil 22.

In colorectal cancers, recent multivariate analyses indicate that loss of CDX2 expression is associated with less‐differentiated carcinomas and advanced cancer stages 12, 24. Loss of CDX2 expression is observed in poorly differentiated mucinous adenocarcinoma of the ovary. MDR1 loss induced by CDX2 suppression could exert a beneficial influence on patient survival by reducing drug resistance 11. However, since CDX2 loss could contribute to aggressive tumor behavior considering the roles of CDX2 in promoting cellular differentiation and inhibiting proliferation 13, it remains unclear whether CDX2 loss plays a critical role in ovarian cancer progression. In colorectal cancer cell lines, activation of CDX2‐induced *MDR1* transcription even in the presence of protein synthesis inhibitors, and four putative CDX2‐binding sites in a 4 kb region upstream of the *MDR1* transcription start site were identified 11, 25. Reporter gene analysis showed that two of these elements were critical, and subsequent ChIP assays showed that CDX2 directly binds to this MDR1 promoter region 11. Thus, CDX2 directly regulates *MDR1* expression in colorectal cancer cells. However, we have not yet confirmed whether CDX2 directly regulates MDR1 expression in ovarian mucinous adenocarcinoma cell lines. There are only few cell lines that could be examined in our study, as the establishment of ovarian mucinous adenocarcinoma cell lines is technically challenging. Therefore, we could only conduct an siRNA experiment using a single cell line (OMC‐1). It is possible that other factors besides CDX2 are required for activation of *MDR1* transcription in certain settings, such as in MN‐1 cells, as MDR1‐positive ovarian mucinous adenocarcinoma cell lines (namely MN‐1) did not express CDX2 mRNA or protein. To date, additional factors regulating MDR1 expression have been proposed, including Y‐box‐binding protein 1 (*YBX‐1*) 26, *SP1* and early growth response element 1 (*EGR1*) 27, NF‐kB (*NFKB1*) 28, p53 29, and *COX2* 30.

The expression of MDR1 is one of markers for cancer stem cells. Besides MDR1, REG IV has also been evaluated as a marker for cancer stem cells 31. Several studies have investigated the functions of REG IV proteins. Results have shown that these proteins increase cell growth via activation of EGF receptor 32, inhibit apoptosis 32, promote metastasis in mouse models, and promote resistant to certain chemotherapeutic compound, such as 5‐fluorouracil (5‐FU) 33. Thus, therapies that target REG IV are thought to be potentially effective. In addition, REG IV is secreted into the blood by cancer cells, so they could serve as a tumor marker for assessing the possibility of cancer using blood tests 33. Additionally, a study on gastric cancer demonstrated that CDX2 regulates REG IV 34. In ovarian mucinous adenocarcinoma, our immunohistochemical staining results suggested a correlation between CDX2 and REG IV expression (data not shown). Clearly, it will be of some interest in future studies to analyze the mechanism where CDX2 regulates REG IV.

The current standard combination chemotherapy for epithelial ovarian cancer is carboplatin and paclitaxel 35. However, the effect of chemotherapeutic drugs is still limited in ovarian mucinous adenocarcinoma 2, 3, 4, 5, 6. We found that expression of CDX2 correlated with MDR1 expression and the degree of differentiation of ovarian mucinous adenocarcinoma cells. In addition, we showed that MDR1 protein expression was related to resistance to certain chemotherapeutic compounds, such as paclitaxel. If elevated MDR1 expression induced by CDX2 is associated with resistance to chemotherapeutic agents, there could be value in inhibiting CDX2 expression in addition to MDR1 inhibitors. Therefore, drug resistance and a poor prognosis could be improved by regulating CDX2 and MDR1. Moreover, CDX2 is a transcription factor that participates in inducing the differentiation of intestinal epithelial cells. Thus, in addition to inhibitors, it could be beneficial to take this property into consideration when examining chemotherapy regimens for colon cancer. Combination chemotherapy of oxaliplatin and 5‐FU has been shown to have positive effects at the cell line 36. In the future, CDX2 could serve as a therapeutic marker alongside MDR1.

In conclusion, our findings demonstrated that MDR1 expression is regulated by CDX2 and is related to drug resistance in ovarian mucinous adenocarcinoma. Although further studies on MDR1 function and its regulation by CDX2 are required to elucidate the complex drug resistance mechanism, our results demonstrate the potential of novel chemotherapy regimens based on CDX2 status and MDR1 expression in ovarian mucinous adenocarcinoma.

## Conflict of interest

None declared.

## Supporting information


**Table S1.** CDX2 and MDR1 expression is coupled in human ovarian cancer (*n* = 53).

**Table S2.** Coexpression of CDX2 and MDR1 in mucinous ovarian cancer (*n* = 14).
