# Supplementary material for: Regulation of multidrug resistance 1 expression by CDX2 in ovarian mucinous adenocarcinoma
Source: Cancer Med. 2016 Apr 6;5(7):1546–55. doi: 10.1002/cam4.697 (PMC4944882; doi:10.1002/cam4.697)
Supplement: Supplementary file 1 — Table S1. CDX2 and MDR1 expression is coupled in human ovarian cancer (n = 53). [file CAM4-5-1546-s001.docx]

| **Table S1.** CDX2 and MDR1 expression is coupled in human ovarian cancer (n=53) | | | | |
| --- | --- | --- | --- | --- |
|  | | MDR1 expression | | *P*-value |
|  |  | positive | negative |  |
| CDX2 expression | positive | 8 | 0 | < 0.001 |
|  | negative | 13 | 32 |  |
