# Supplementary material for: Regulation of multidrug resistance 1 expression by CDX2 in ovarian mucinous adenocarcinoma
Source: Cancer Med. 2016 Apr 6;5(7):1546–55. doi: 10.1002/cam4.697 (PMC4944882; doi:10.1002/cam4.697)
Supplement: Supplementary file 2 — Table S2. Coexpression of CDX2 and MDR1 in mucinous ovarian cancer (n = 14). [file CAM4-5-1546-s002.docx]

| **Table S2.** Coexpression of CDX2 and MDR1 in mucinous ovarian cancer (n=14) | | | | |
| --- | --- | --- | --- | --- |
|  | | MDR1 expression | | *P*-value |
|  |  | positive | negative |  |
| CDX2 expression | positive | 7 | 0 | 0.02 |
|  | negative | 2 | 5 |  |
